# Permanent Pacing in Patients with Recurrence of Symptoms and Relapse of Left Ventricular Obstruction at Midcavity Level after Alcohol Septal Ablation

**DOI:** 10.1155/2012/757501

**Published:** 2012-02-19

**Authors:** Vasil Velchev, Arman Postadzhiyan, Dobri Hazarbasanov, Bojidar Finkov

**Affiliations:** Cardiology Department, St. Anna University Hospital, 1709#, Sofia, Bulgaria

## Abstract

Treatment of symptom recurrence after initially successful alcohol septal ablation (ASA) in hypertrophic obstructive cardiomyopathy (HOCM) when accompanied by relapse of intracavitary left ventricular pressure gradient (LVG) is guided by the underlying mechanism. We describe our experience with permanent pacing in three patients with relapse of both LVG and symptoms 7 to 12 months after successful ASA. Even though pressure gradient recurrence was observed at midventricular level, we were able to achieve symptomatic improvement and LVG reduction after right ventricular apex pacing in all three cases. The effect on symptoms was long lasting—the 6-month followup echo-stress tests confirmed good exercise capacity and lack of provocable LVG. We found pacing to be a safe and effective treatment option in this clinical scenario. Based on our overall observations, we propose pacing as a niche treatment for patients with recurrence of LVG at midventricular level after ASA.

## 1. Introduction

Alcohol septal ablation (ASA) is becoming a popular treatment choice for symptomatic patients with hypertrophic obstructive cardiomyopathy (HOCM) and resting left ventricular outflow tract obstruction gradient (LVOTG) above 50 mmHg.The recurrence of symptomatic LVOTG is approximately 10 percent in the first 2 years after ASA [1, 2]. Current hypotheses to explain this recurrence include possible incomplete remodeling of the septum due to imprecise location of the iatrogenic lesion, a small infarct size because of suboptimal ethanol tissue concentration and an underestimation of existent surgical pathology such as massive calcification of the mitral ring [2–4]. To the best of our knowledge, late recurrence of symptoms due to “migration” of obstruction to midcavity level following ASA has not been described in the literature.

Therapeutic options for symptomatic patients with high residual LVOTG after ASA include surgery, repeat ASA, permanent pacing, and medical therapy. It is reasonable to speculate that permanent pacing has a synergistic effect on LVOTG after ASA and could be attempted before deciding on a repeat ASA or surgical myectomy.

 Here we present our experience with permanent pacing in three patients with HOCM who had recurrence of symptoms and LVG after initially successful alcohol septal ablation.

Sixty-one patients with HOCM who remained symptomatic despite medical treatment underwent alcohol septal ablation at our institution between October 2004 and October 2007. Only patients with typical systolic anterior motion (SAM) and invasively measured LVOTG >50 mmHg at rest or at provocation were included. Our technique is described in a separate article and does not differ considerably from the one described in the literature [5]. One milliliter of 95% ethanol per cm of septum thickness was injected through an OTW balloon catheter after positive pressure reduction test. If equivocal results were observed, echo contrast was injected through an inflated balloon to delineate the part of the septum supplied by the septal branch. Long-term success was defined as resting LVOTG less than 20 mmHg and/or 50% reduction of resting and provoked LVOTG matched by relief of the symptoms. Success was achieved in 53 patients (87%) who were followed for a median of 21 months (3–35). No mortality was observed at 30 days, 2 patients died at 9 and 18 months, and the causes of death were sudden cardiac death and terminal heart failure. Recurrence of LVG and symptoms were observed in 10 patients during the follow-up period. Six patients were successfully treated with repeat ASA because of echo findings of SAM recurrence and three patients received permanent right ventricular pacing. One patient refused further treatment and died from heart failure.

**Figure 1:**
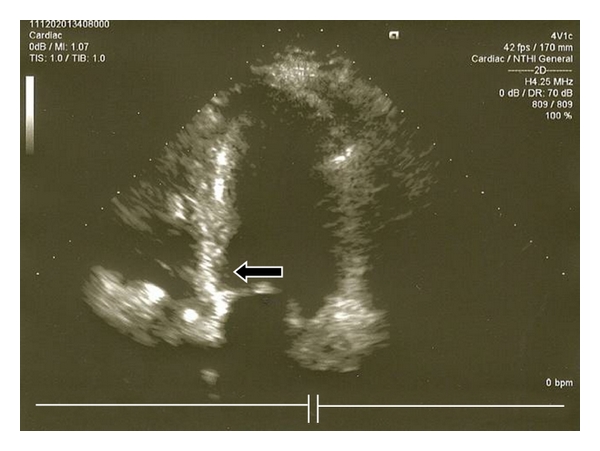
Control echo of patient N1 6 months after ASA. Thinning of basal septum is clearly visible (arrow).

We focus on three patients who share a similar clinical scenario: late relapse of symptoms due to dynamic obstruction at midcavity level following ASA despite remodeling and thinning of the basal septum. All patients underwent initial ASA at our institution due to effort-limiting angina refractory to beta blockers. All three had a similar underlying LV morphology with echocardiography revealing thickening of the whole septum, typical SAM leading to outflow tract obstruction, and eccentric mitral regurgitation. The septal thickness at anterior mitral leaflet contact was 21, 24, and 20 mm, respectively for, patients 1, 2, and 3. Six months after ASA the result was judged as excellent based on the disappearance of symptoms and virtual abolishment of LVOTG at rest echo. Thinning and akinesia of the basal septum was evident as a proof of well-placed scar (Figure 1).

**Figure 2:**
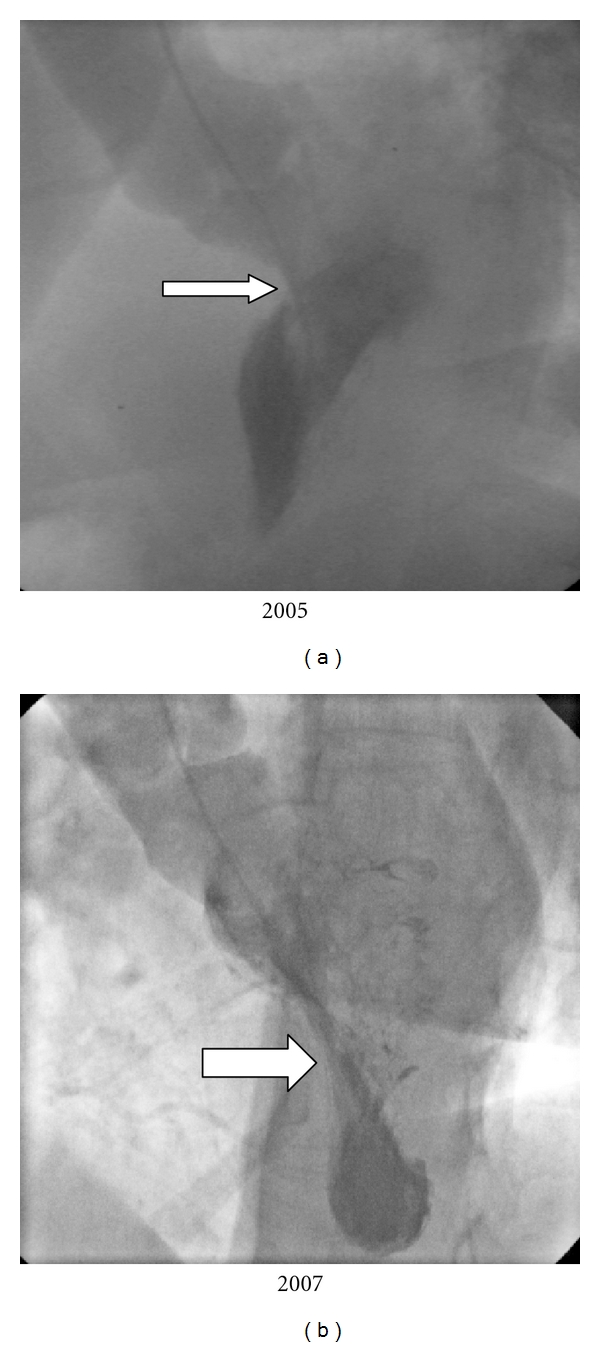
Patient N1 presented with syncope and 50 mmHg gradient at midcavity level 2 years after ASA. LV-gram before ASA (a) shows hyperkinetic LV and typical SAM (arrow). LV-gram at 26-month followup (b) is remarkable for the typical dumbbell shape in systole with formation of distal akinetic camera (thick arrow).

Patient N1 was a 54-year-old man who presented with syncope 26 months after ASA and echocardiography at rest showed left ventricular systolic gradient of 42 mmHg at midcavity level and no SAM. At the index procedure, 2.2 mL of alcohol were injected in the 1st septal perforator artery with complete resolution of the LVOTG and reduction of the mitral regurgitation grade. Similarly, patients N2 (a 42-year-old woman) and N3 (a 61-year-old man) presented 7 and 23 months after successful ASA with recurrence of effort angina. Invasive assessment of all three patients revealed intracavitary pressure gradient at the level of the papillary muscles with formation of a distal akinetic chamber (Figure 2). In all three patients, echocardiography demonstrated marked scarring of the basal septum as expected late after ASA (septum thickness 16, 18, and 15 mm, resp., for patients 1, 2, and 3) and relatively hypokinetic apex with systolic kissing of the midsegments of LV. In fact, subaortic obstruction was effectively converted to a midventricular one. After failure to control symptoms with beta blockers, we decided to proceed with permanent pacemaker implantation.

 In two patients permanent DDD mode pacemakers were implanted with passive fixation electrodes positioned at RV apex and right atrial appendage. In one patient (N1) VVI pacemaker was implanted after AV node ablation for high-rate atrial fibrillation. The RV electrode was implanted aiming at the pig-tail catheter positioned at LV apex. No estimation of the temporary pacing effect on the LVOTG was performed. The longest PV interval was adjusted to achieve full ventricular capture. Ventricular pacing of 99 percent was achieved in all patients (PV interval of 160 msec for DDD pacemakers).

All three patients had echo at rest and after a stress test at the 6-month followup The resting LVGs measured by Doppler echocardiography before ASA, at symptom recurrence and at 6 months after pacing are shown in Table 1.

All three patients were able to achieve 5 METS on a modified Bruce protocol at 6-month followup, and echo detected no LVOTG (defined as velocity >2 m/s a) at rest and after exercise. Functional class significantly improved from NYHA III prior to treatment to NYHA II after-pacing. This result was sustained for 6 months of followup.

## 2. Discussion

Despite promising early results, permanent pacing is falling out of favor as a first-line therapy for drug-resistant HOCM due to its lower success rate demonstrated in randomized studies as compared to myectomy and ASA [6, 7]. The reasons for these unsatisfactory results are not clear. A possible explanation is the inability to achieve full reverse excitation of the septum in a significant proportion of the patients due to short intrinsic AV interval or technical factors such as the inability to place the electrode at the true RV apex. Even if some patients do respond to ASA in terms of long-lasting symptom relief, predictors of success have yet to be definitively identified [8].

The factors leading to ASA failure are also far from clear. On the basis of studies that followed patients with MRI after ASA, it can be extrapolated that in a significant number of ablated patients the localization of the infarct is away from the zone of contact between the anterior mitral leaflet and the septum [4]. Other investigators suggest on the basis of postmortem histology that, for unknown reasons, even a properly located lesion can lead to patchy instead of dense fibrosis with only mild septum thinning [3]. In both cases, the remodeling of the septum does not lead to outflow tract widening.

In contrast to other investigators, we have observed several cases of recurrence of LVOTG and symptoms well after the first year of followup [9]. This might be related to the selection of patients or to a technical factor although we have not found major differences between our technique and the one already published in the literature. In the proportion of our patients, distal displacement of the level of obstruction due to remodeling of the basal septum led to transformation of subaoric stenosis into hourglass left ventricle. To the best of our knowledge, no such case has been previously described in the literature.

Choice of treatment after ASA failure appears to depend on the underlying mechanism, with drug therapy rarely effective in our experience. The small number of cases of ASA failures makes it highly unlikely that a comparative study of different treatment modalities can be conducted. Repeat ASA appears to be a logical choice if there is relapse of LVOTG, SAM and no evidence of effective remodeling of the septum. Due to the lack of reliable criteria for selection of responders, current guidelines recommend pacing in HOCM only in case of coexistent bradycardia/conduction disease or indications for ICD placement [2, 8]. There is also agreement that patients with preexisting bundle branch block who are candidates for ASA or surgery should receive a pacemaker if the risk of procedure-related AV block is high [10]. In our institution, we consider pacing as a first-line treatment for midcavity obstruction because it is relatively non-invasive and at least 1 publication suggests that it is efficacious [11]. Overall, we have found permanent pacing to be a good solution even when midcavity obstruction manifested late after ASA.

## 3. Conclusions

We found a specific pattern of progression of HOCM after successful ASA. In our experience permanent RV pacing appears to be a safe and effective treatment option with no major drawbacks. It could be proposed as a niche treatment for patients with recurrence of LVG at midventricular level after ASA.

## Figures and Tables

**Table 1:** Dynamics of LVOTG as measured by echocardiography.

Patient no.	LVG at rest before ASA (mmHg)	LVG at rest 6 mo after ASA (mmHg)	LVG at recurrence of symptoms ASA (mmHg)	LVG 6 mo after pacing (mmHg)
1	80	>16	42	>16
2	55	>16	60	>16
3	50	>16	30	>16
